# Draft Genome Sequence of *Mycobacterium tuberculosis* Clinical Strain W06, a Prevalent Beijing Genotype Isolated in Taiwan

**DOI:** 10.1128/genomeA.01460-15

**Published:** 2015-12-10

**Authors:** Yu-Chieh Liao, Tze-Tze Liu, Jia-Ru Chang, Yih-Yuan Chen, Hsin-Hung Lin, Chih-Hao Hsu, Ching-Yen Lin, Ying-Chih Lo, Horng-Yunn Dou

**Affiliations:** a National Institute of Infectious Diseases and Vaccinology, National Health Research Institutes, Zhunan, Miaoli, Taiwan; b Division of Biostatistics and Bioinformatics, Institute of Population Health, National Health Research Institutes, Zhunan, Miaoli, Taiwan; c Genome Research Center, National Yang-Ming University, Taipei, Taiwan

## Abstract

*Mycobacterium tuberculosis* strain W06, analyzed by molecular methods, was classified as a modern Beijing *M. tuberculosis* strain, the most predominant strain in Taiwan. To our knowledge, this is the first draft genome announcement of a Beijing *M. tuberculosis* strain in Taiwan.

## GENOME ANNOUNCEMENT

Tuberculosis (TB) remains a leading notifiable infectious disease in Taiwan. Based on the epidemiological surveillance reported by Chen and Mokrousov et al., the Beijing genotype is a prevalent *M. tuberculosis* strain and is often associated with highly virulent, multiple-drug resistance and is endemic throughout Asia and worldwide (1–3). Beijing lineage is a predominant genotype, especially in northern Taiwan, whereas the East-African Indian (EAI) lineage is particularly prevalent in southern Taiwan (4, 5). Previous studies indicated that the modern Beijing strain, W06, when processing the intact RD150 and RD142 region, induced very low levels of proinflammatory cytokine compared to the ancient Beijing lineage (6). The differences in immune responses elicited by *M. tuberculosis* strains may explain why modern Beijing genotypes are more prevalent than other genotypes in Taiwan.

Here we report the complete genome sequence in Taiwan of a clinical isolate of *M. tuberculosis* strain W06, isolated at Wan-Qiao branch, Taichung Veterans General Hospital, from human sputum of a patient confirmed as having TB with positive sputum microscopy and culture. The genomic DNA of strain W06 was extracted from cultured cells as described previously (7) and was sequenced using a MiSeq platform (Illumina, USA). Strain W06 was also analyzed by spoligotyping (000000000003771) and mycobacterial interspersed repetitive unit-variable number tandem repeat (MIRU-VNTR) typing (424522332517333474433582) and was confirmed as a member of the Beijing family. This study was approved by the Human Ethics Committee of the National Health Research Institutes, Taiwan (code EC1010804-E). Because of the retrospective nature of the study, routine collection of clinical data in daily practice, and dislinkage of personal information, the requirement to obtain informed consent was waived by our institutional review board.

A whole-genome shotgun library and an 8-kb-span paired-end library were constructed using a GS Titanium rapid library preparation kit (Roche) and Roche’s paired-end rapid library preparation method, respectively, followed by sequencing on a GS Junior System (Roche) according to the manufacturer’s protocol. The whole-genome shotgun reads and paired-end reads were assembled using Newbler v. 2.6 and yielded 142 large contigs and 2 scaffolds with approximately 27-fold genome coverage of 4.3-Mb genome size. Additional Illumina MiSeq reads were generated from a shotgun library constructed using a TruSeq DNA PCR-free sample preparation kit (Illumina) and assembled into 132 contigs using CLC Genomics Workbench v7.5 (CLC Bio). These Illumina contigs were used to close gaps or extend contig ends in the GS assembly using Phred/Phrap/Consed (8). Certain gaps were filled by PCR and Sanger sequencing. The final assembly had 4,505,137 bp in 5 contigs with contig sizes of 2,615,264 bp, 1,207,678 bp, 566,923 bp, 105,814 bp, and 9,458 bp.

### Nucleotide sequence accession numbers.

This whole-genome shotgun project has been deposited at GenBank under the accession number LHCK00000000. The version described in this paper is in the first version, LHCK01000000. The BioProject ID is PRJNA291305.
